# A group VII ethylene response factor gene, *ZmEREB180*, coordinates waterlogging tolerance in maize seedlings

**DOI:** 10.1111/pbi.13140

**Published:** 2019-05-14

**Authors:** Feng Yu, Kun Liang, Tian Fang, Hailiang Zhao, Xuesong Han, Manjun Cai, Fazhan Qiu

**Affiliations:** ^1^ National Key Laboratory of Crop Genetic Improvement Huazhong Agricultural University Wuhan China

**Keywords:** adventitious root, ERFVIIs, ethylene, maize, natural variation, reactive oxygen species, waterlogging stress

## Abstract

Group VII ethylene response factors (*ERFVIIs*) play important roles in ethylene signalling and plant responses to flooding. However, natural *ERFVII* variations in maize (*ZmERFVIIs*) that are directly associated with waterlogging tolerance have not been reported. Here, a candidate gene association analysis of the *ZmERFVII* gene family showed that a waterlogging‐responsive gene, *ZmEREB180*, was tightly associated with waterlogging tolerance. *ZmEREB180* expression specifically responded to waterlogging and was up‐regulated by ethylene; in addition, its gene product localized to the nucleus. Variations in the 5ʹ‐untranslated region (5ʹ‐UTR) and mRNA abundance of this gene under waterlogging conditions were significantly associated with survival rate (SR). Ectopic expression of *ZmEREB180* in *Arabidopsis* increased the SR after submergence stress, and overexpression of *ZmEREB180* in maize also enhanced the SR after long‐term waterlogging stress, apparently through enhanced formation of adventitious roots (ARs) and regulation of antioxidant levels. Transcriptomic assays of the transgenic maize line under normal and waterlogged conditions further provided evidence that *ZmEREB180* regulated AR development and reactive oxygen species homeostasis. Our study provides direct evidence that a *ZmERFVII* gene is involved in waterlogging tolerance. These findings could be applied directly to breed waterlogging‐tolerant maize cultivars and improve our understanding of waterlogging stress.

## Introduction

Waterlogging is one of the most important abiotic stresses affecting crop growth, development and yield. Flooding events associated with global climate change have occurred increasingly since the start of this century (Bailey‐Serres *et al*., 2012; Hirabayashi *et al*., 2013). Maize (*Zea mays* L.) frequently encounters waterlogging stress during its life cycle due to poor drainage and/or long periods of rainfall (Visser *et al*., 2003). Waterlogged soil results in reduced levels of oxygen in plant tissues and less gas diffusion between cells (Voesenek and Bailey‐Serres, 2013), which restricts mitochondrial respiration and decreases soil pH (Fukao and Bailey‐Serres, 2004; Setter *et al*., 2009). The accumulation of reactive oxygen species (ROS) that cause oxidative damage to plants is also enhanced in conditions of prolonged low‐oxygen stress (Shabala, 2011).

To survive and regulate different responses under waterlogging stress, plants modulate numerous morphological, transcriptional and metabolic changes (Bailey‐Serres and Colmer, 2014; Bailey‐Serres and Voesenek, 2008; Lee *et al*., 2011; Nanjo *et al*., 2011; Narsai *et al*., 2009; Zou *et al*., 2010). Anaerobic pathways, such as ethanolic fermentation and glycolysis coupled with nicotinamide adenine dinucleotide (NAD) regeneration, respond to fulfil adenosine triphosphate (ATP) needs under waterlogging stress, and adaptive traits such as aerenchyma and adventitious root formation are induced to promote gas diffusion (Colmer and Voesenek, 2009). In addition, endogenous antioxidant enzymes and nonenzymatic molecules are up‐regulated to counteract the deleterious effects of ROS (Apel and Hirt, 2004; Mittler *et al*., 2004).

Research on rice has revealed that two ethylene‐mediated opposite signalling pathways are involved in submergence tolerance (Voesenek and Bailey‐Serres, 2015). *SUBMERGENCE 1A* (*SUB1A*) confers submergence tolerance in rice (Xu *et al*., 2006). Ethylene‐induced *SUB1A* increases the accumulation of gibberellin (GA) signalling repressors to inhibit the transcription of GA‐inducible genes, limiting GA‐mediated starch breakdown, elongation growth and leaf senescence and enhancing the use of carbohydrate reserves (Fukao and Bailey‐Serres, 2008; Fukao *et al*., 2006, 2012; Hirano *et al*., 2012). Tolerant genotypes with the *SUB1A* allele show up‐regulation of mRNAs encoding antioxidant enzymes during submergence (Jung *et al*., 2010; Mustroph *et al*., 2010). By contrast, the induction of *SNORKEL1* (*SK1*) and *SK2* by ethylene in deepwater rice varieties promotes internode elongation by increasing the accumulation of active GA, which also fits within the hormonal hierarchy (Ayano *et al*., 2014; Hattori *et al*., 2009; Raskin and Kende, 1984). Similar survival strategies in response to flooding have also been found in other species, such as *Rumex* species (Bailey‐Serres and Voesenek, 2008; Benschop *et al*., 2005). Moreover, studies on *Arabidopsis* have shown that petiole elongation and leaf hyponastic growth are adaptive responses to submergence in partial or complete darkness and likely occur via an ethylene‐dependent process (Lee *et al*., 2011). Recently, the gene determining leaf gas films [*LEAF GAS FILM1* (*LGF1*)] has been shown to enhance underwater photosynthesis while contributing to submergence tolerance in rice via regulating C30 primary alcohol synthesis (Kurokawa *et al*., 2018). These studies provide the basis for a better understanding of how plants adapt to excess water environments.

The ethylene response factor (ERF) family is a large gene family of plant‐specific transcription factors characterized by a single DNA‐binding APETALA2 (AP2)/ethylene‐responsive element‐binding protein domain (Licausi *et al*., 2013; Nakano *et al*., 2006). A subgroup of this family, the ERFVIIs, is characterized by several other motifs in addition to the AP2 domain (Nakano *et al*., 2006). ERFVIIs control flooding responses and low‐oxygen tolerance in several plant species (Gibbs *et al*., 2015). *Arabidopsis* encodes five ERFVIIs, including two known hypoxia‐responsive ERFs (HRE1 and HRE2) that directly affect anaerobic responses (Licausi *et al*., 2010). The survival rate of the double‐knockout mutant *hre1hre2* in *Arabidopsis* is markedly lower in comparison with the wild type, whereas overexpressed *HRE1* exhibits improved tolerance to anoxia (0% oxygen), positively regulating core hypoxia‐responsive gene expression (Hess *et al*., 2011; Licausi *et al*., 2010). Another ERFVII in *Arabidopsis*,* RAP2.2*, has a similar function of flooding response (Hinz *et al*., 2010). *RAP2.12* is highly homologous to *RAP2.2* and is up‐regulated in leaves under hypoxic conditions. The RAP2.12 protein is anchored to membrane‐bound ACYL‐COA BINDING PROTEIN1 (ACBP1) under aerobic conditions, but it is released and translocated to the nucleus to activate the expression of hypoxia‐responsive genes at the onset of hypoxia (Licausi *et al*., 2011). Thus, there is strong evidence that ERFVIIs in *Arabidopsis* are directly involved in anoxia/hypoxia signalling and regulate survival ability under stress conditions. Notably, three genes (*SUB1A*,* SK1* and *SK2*) conferring submergence tolerance in rice also belong to the ERFVII family (Hattori *et al*., 2009; Xu *et al*., 2006). Furthermore, the flooding responses of two related *Rumex* spp. species from contrasting hydrological environments may also be regulated by ERFVIIs (van Veen *et al*., 2014).

Maize is sensitive to waterlogging stress. Waterlogging severely affects approximately 18% of the land in south and south‐east Asia and annually results in production losses of 25%–30% (Zaidi *et al*., 2010). Investigations of maize and its tolerant ancestor *Zea nicaraguensis* in waterlogged conditions have revealed that enhanced formation of aerenchyma and induction of barriers to radial oxygen loss in adventitious roots contribute to waterlogging tolerance (Abiko *et al*., 2012; Watanabe *et al*., 2017). Other research has shown that programmed cell death (PCD) in the root cortex and the formation of aerenchyma and adventitious roots are the most adaptive traits in maize (Thirunavukkarasu *et al*., 2013; Zhai *et al*., 2013). Increasing the oxygen available to waterlogged organs is thus vital for maize to adapt to waterlogged conditions, but the regulation mechanisms of these adaptive traits in maize are unclear.

In this study, we identified and characterized 19 *ERFVIIs* of maize (*ZmERFVIIs*). The association between genetic variations of each *ZmERFVII* gene and waterlogging tolerance, evaluated in terms of survival rate (SR) under long‐term stress at the seedling stage, was quantified using a candidate gene association strategy and a diverse population of maize consisting of 368 inbred lines from global germplasm. A key candidate gene, *ZmEREB180*, was strongly associated with SR under multiple environments. Variations in the 5ʹ untranslated region (5ʹ‐UTR) of *ZmEREB180* affected its expression in different varieties and showed significant correlation with SR. Functional analysis showed that *ZmEREB180* plays a positive role in maize waterlogging tolerance, seemingly by promoting the formation of adventitious roots and coordinating ROS homeostasis. Our results improve our understanding of the molecular mechanisms in waterlogging responses and accelerate application of marker‐assisted breeding for waterlogging tolerance.

## Results

### Identification and cloning of *ZmERFVII* genes

To identify the group VII ethylene response factors in maize (*ZmERFVIIs*), the predicted protein sequences of 212 AP2 and ethylene‐responsive element‐binding protein (AP2‐EREBPs) genes were downloaded from GrassTFDB in GRASSIUS (Burdo *et al*., 2014). The five *Arabidopsis ERFVII* genes (Nakano *et al*., 2006) were used as queries to search against these sequences, and two signatures (alanine at position 13 and aspartic acid at position 18; Magnani *et al*., 2004) of ERFVII genes were further confirmed manually. Finally, 19 *ZmERFVIIs* were identified. We cloned these genes from the B73 maize inbred line and found that they were identical to the annotated sequences. These genes are located in all chromosomes except chromosomes 8 and 10 (Figure S1, Table S1). Whereas *ZmEREB179* had two introns, the other 11 and seven genes had one and zero introns, respectively (Figure S2). Motif analysis revealed numerous conserved motifs in addition to the AP2/ERF domain (Figure S2). The conserved N‐terminal motif (CM1) divides ERFVIIs into subgroup a and subgroup b depending on whether they contain CM1 (Bailey‐Serres *et al*., 2012). Among the *ZmERFVIIs*, 12 and 7 belonged to subgroup a and subgroup b, respectively. By contrast, none of the 5 *ERFVIIs* in *Arabidopsis* and only 1 of the 15 *ERFVIIs* in rice belong to subgroup b (Bailey‐Serres *et al*., 2012; Nakano *et al*., 2006), indicating that *ZmERFVIIs* not only include more family members than in *Arabidopsis* and rice but also exhibit more sequence variability, implying a greater diversity of functions.

### Association analysis of natural variation in *ZmERFVIIs* with maize waterlogging tolerance

From previously reported transcriptomic sequencing of a maize panel consisting of 368 inbred lines, high‐quality SNP markers with a minor allele frequency of ≥5% (Fu *et al*., 2013) were used to characterize the DNA polymorphism of 19 *ZmERFVII* genes. Among these *ZmERFVII* genes, all were found to be polymorphic; on average, 31.95 SNP markers were identified in each gene, and *ZmEREB102* exhibited the most polymorphic markers (Table 1). The waterlogging tolerance of each inbred line was investigated, and the survival rate (SR) of each genotype was determined. The SR of inbred lines exhibited wide phenotypic variations that ranged from 0% to 100%, mainly distributed between 30% and 60% (Figure S3). Combining the genotypic SNP data from 19 *ZmERFVII* genes with SR phenotypes in three different environments and best linear unbiased prediction (BLUP) data, an association analysis was conducted to quantify significant associations between SNP and traits. Nine genes were significantly associated with SR in at least one environment (*P < *0.01), of which *ZmEREB179* and *ZmEREB180* were identified in multiple environments (Table 1). Only *ZmEREB180* was associated with waterlogging tolerance in this panel at *P < *0.001 (Table 1).

**Table 1:** Association analysis of natural variations in *ZmERFVIIs* genes with waterlogging tolerance at the seedling stage in the maize diversity panel

Gene*	Chr.†	Number of polymorphism markers	*P* < 1.0E‐2				*P* < 1.0E‐3			
			EXP1‡	EXP2‡	EXP3‡	BLUP‡	EXP1	EXP2	EXP3	BLUP
*ZmEREB179*	1	44	2	0	2	4	0	0	0	0
*ZmEREB180*	1	39	5	8	2	7	3	4	2	4
*ZmEREB181*	1	11	0	0	2	0	0	0	0	0
*ZmEREB182*	1	38	0	0	3	0	0	0	0	0
*ZmEREB172*	1	21	0	0	0	0	0	0	0	0
*ZmEREB167*	1	36	0	0	0	0	0	0	0	0
*ZmEREB211*	2	21	0	6	0	0	0	0	0	0
*ZmEREB202*	2	56	0	0	0	0	0	0	0	0
*ZmEREB210*	2	12	0	0	0	3	0	0	0	0
*ZmEREB193*	3	46	0	0	0	0	0	0	0	0
*ZmEREB14*	4	4	0	0	1	0	0	0	0	0
*ZmEREB7*	4	19	0	0	0	3	0	0	0	0
*ZmEREB90*	5	46	0	0	0	0	0	0	0	0
*ZmEREB139*	5	10	0	0	0	0	0	0	0	0
*ZmEREB69*	6	33	0	0	0	0	0	0	0	0
*ZmEREB200*	7	11	0	0	0	0	0	0	0	0
*ZmEREB116*	7	22	0	0	0	0	0	0	0	0
*ZmEREB102*	7	88	1	0	0	0	0	0	0	0
*ZmEREB160*	9	50	0	0	0	0	0	0	0	0

* Gene name from GrassTFDB in GRASSIUS.

† Chromosome.

‡ EXP1, EXP2, EXP3 and BLUP represent environment 1, environment 2, environment 3 and BLUP data, respectively.

**Figure 1:**
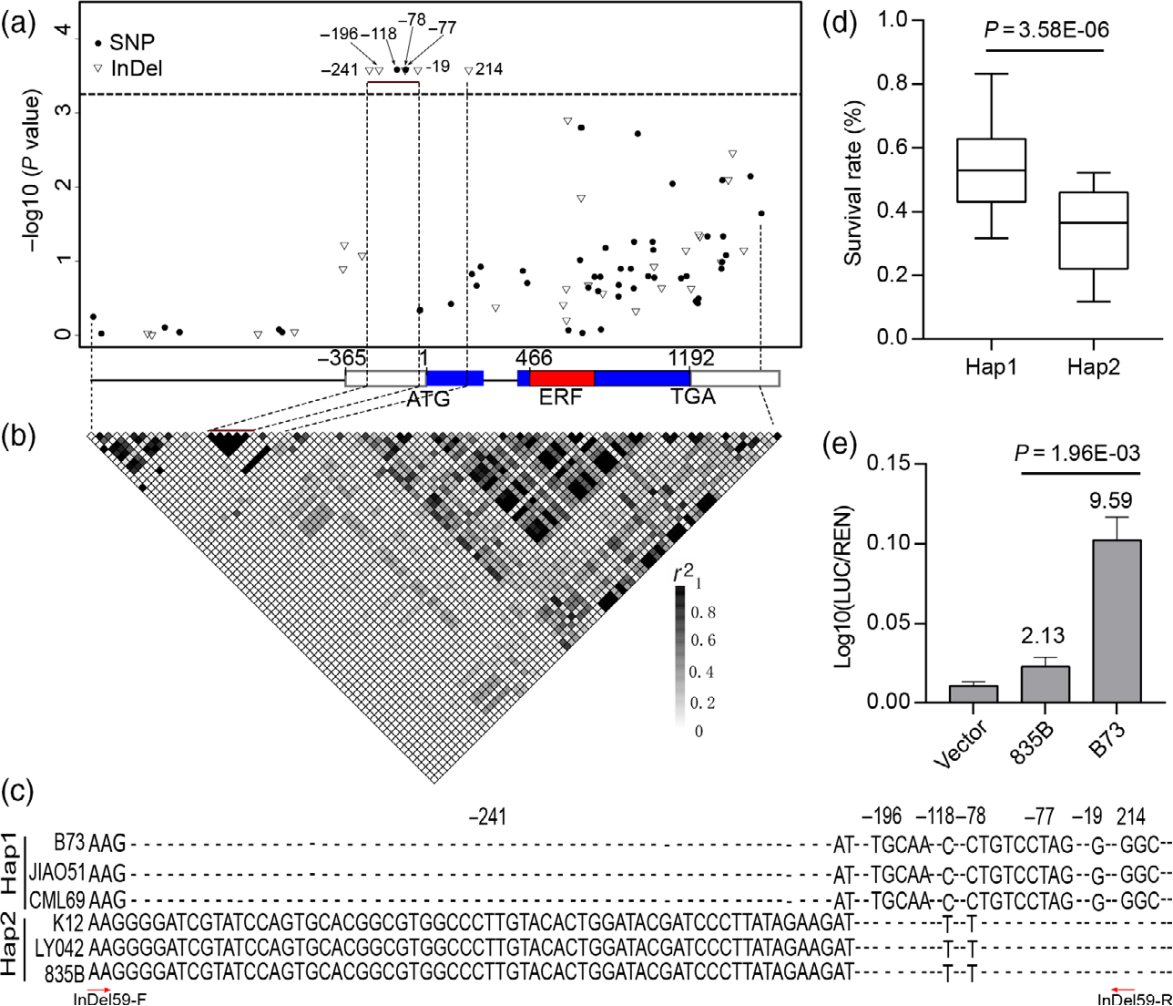
Natural variations in *ZmEREB180* are significantly associated with waterlogging tolerance in maize. (a) Association analysis of genetic variation in *ZmEREB180* with waterlogging tolerance in maize. A schematic diagram of the 3.1‐kb genomic fragment, including 1170‐bp promoter, 365‐bp 5ʹ‐UTR, 397‐bp 3ʹ‐UTR and the protein coding region, is presented on the *x*‐axis. The location of the initiation codon (ATG) is marked as ‘1'. *P* values are shown on a −log10 scale. (b) The pattern of pairwise LD of DNA polymorphisms of *ZmEREB180*. (c) Haplotypes of *ZmEREB180* in maize genotypes B73, JIAO51, CML69, K12, LY042 and 835B. Seven DNA polymorphisms are significantly associated with maize waterlogging tolerance. The red arrows represent the location of the InDel59 primer. (d) Comparison of survival rate (SR) for Hap1 and Hap2 in 248 resequenced lines. (e) Comparison of effects of two types of promoter (with 5ʹ‐UTR) sequence (B73 and 835B) on *LUC* reporter gene expression. The numbers in the chart represent the fold increase compared with the negative control (Vector). Vector denotes the empty vector with mini35S promoter. Data represent means ± SD of three independent replicates. Hap1 and Hap2 represent tolerant and sensitive alleles, respectively. *N* is the genotype number of the two alleles. Statistical significance was determined by analysis of variance (ANOVA). Hap, haplotype; LUC, Firefly luciferase; REN, Renilla luciferase.

To identify genetic variations in *ZmEREB180*, a 3.1‐kb genomic sequence containing *ZmEREB180* was resequenced in 248 inbred lines randomly selected from the 368 line association panel. In total, 58 single nucleotide polymorphisms (SNPs) and 29 insertion/deletions (InDels) were detected. We again analysed the association of each polymorphism with BLUP data using the compressed mixed linear model (cMLM) and calculated the pairwise linkage disequilibrium (LD) of these polymorphisms (Figure 1). Six variants, InDel‐241 (59 bp), InDel‐196 (5 bp), SNP‐118, SNP‐78, InDel‐77 (8 bp) and InDel‐19 (1 bp) in 5ʹ‐UTR, and one variant (InDel214, 3 bp) in the first exon were significantly associated with maize waterlogging tolerance (SR; *P < *3.67 × 10^−4^) and completely mapped within an LD block (Figure 1a,b). These resequenced genotypes were classified into two haplotype (Hap) groups based on the significant variants (Figure 1c). Hap1 had a significantly higher SR than Hap2 (*P *=* *3.58 × 10^−6^; Figure 1d), and Hap1 was therefore designated as the favourable/tolerant allele, explaining 15.6% of the phenotypic variation. The amplicon of the primer (InDel59) covered the significant loci in the 5ʹ‐UTR, which should distinguish between Hap1 and Hap2 (Figure 1c, Table S2). Notably, the 3 bp of InDel214 in the exon caused an amino acid (glycine) deletion, although InDel214 did not lead to variations in other amino acids. Moreover, the expression of the B73 (Hap1) promoter (with 5ʹ‐UTR)‐driven LUC reporter in maize protoplasts was significantly higher than that driven by the 835B (Hap2) promoter (with 5ʹ‐UTR; Figure 1e). These results demonstrate that 5ʹ‐UTR variants may confer waterlogging tolerance by affecting the expression of *ZmEREB180*.

### Variants in the 5ʹ‐UTR correlate with *ZmEREB180* mRNA abundance

**Figure 2:**
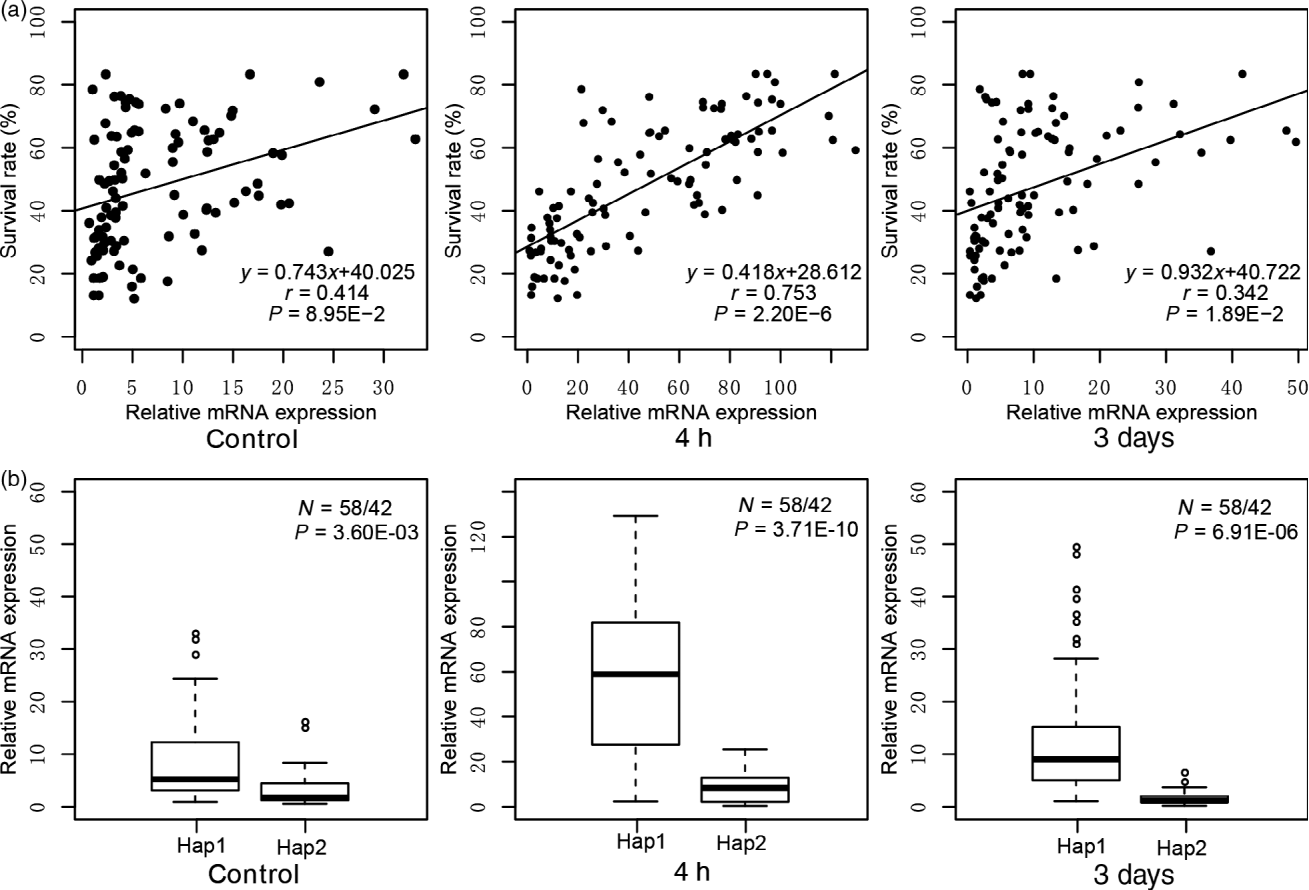
The mRNA abundance of *ZmEREB180*. The relative mRNA abundance of *ZmEREB180* under control, short‐term stress (4 h) and long‐term stress (3 days) was analysed. (a) Correlation between the mRNA abundance of *ZmEREB180* and phenotype of plant survival rate. (b) Comparison of relative mRNA abundance of *ZmEREB180* between Hap1 and Hap2 genotypes.

To examine whether variants in the 5ʹ‐UTR result in altered *ZmEREB180* mRNA abundance among different genotypes, we analysed the mRNA abundance of the *ZmEREB180* gene among 100 inbred lines under well‐watered (control, before waterlogging treatment), short‐term (4‐h) and long‐term (3‐day) waterlogging stress using quantitative reverse‐transcription PCR (qRT–PCR). *ZmEREB180* mRNA abundance was positively correlated with plant SR under both 4‐h and 3‐day waterlogging stress, but there was no significant correlation under well‐watered conditions (Figure 2a). These observations suggest that the increased mRNA abundance in the *ZmEREB180* gene is closely associated with the waterlogging tolerance of maize inbred lines investigated in this study under waterlogging stress. Furthermore, the genotypes with Hap1 exhibited significantly higher mRNA abundance of *ZmEREB180* than those with Hap2 whether the stress was imposed or not, and the mRNA abundance of *ZmEREB180* was up‐regulated in both Hap1 and Hap2 to different extents (Figure 2b), indicating that the mRNA abundance of *ZmEREB180* is also associated with 5ʹ‐UTR variants. Based on these results, we suggest that variations in the 5ʹ‐UTR of *ZmEREB180* that altered its expression level might be the important causal determinants conferring waterlogging stress tolerance in maize seedlings.

### *ZmEREB180* overexpression confers seedling waterlogging tolerance

Considering that *ZmEREB180* expression is positively correlated with waterlogging tolerance of maize seedlings, we performed transgenic assays overexpressing the coding sequence of *ZmEREB180* (from the B73 genotype) in both *Arabidopsis* and maize. For *Arabidopsis*, two independent lines (OE5 and OE8) were used to evaluate the phenotypes after submergence treatment. The transgenic *Arabidopsis* displayed significantly enhanced submergence tolerance in comparison with the wild type (WT), and there were no remarkable morphological changes under normal conditions in transgenic seedlings (Figure S4). After recovery, the fresh weight of aboveground seedlings in transgenic plants was significantly higher than in WT (Figure S4). Moreover, four anaerobic metabolism genes (*AtADH1*,* AtPDC1*,* AtSUS1* and *AtSUS4*; Licausi *et al*., 2010) were significantly up‐regulated in the transgenic plants compared with WT under submerged conditions (4 h), but not significantly altered under normal conditions (0 h; Figure S4), indicating that expression of *ZmEREB180* in *Arabidopsis* enhanced the effect of submerging treatment. The physiological assays of OE5, OE8 and WT demonstrated that MDA content, indicating the degree of oxidative damage, was not significantly different between transgenic lines and WT under normal conditions (Figure S5). However, WT exhibited significantly higher MDA contents after 1 and 2 days of submergence stress. The H_2_O_2_ content was significantly lower in transgenic lines than in WT whether the stress occurred or not (Figure S5), whereas the activity of POD in transgenic lines was significantly higher than in WT after 2 days stress.

**Figure 3:**
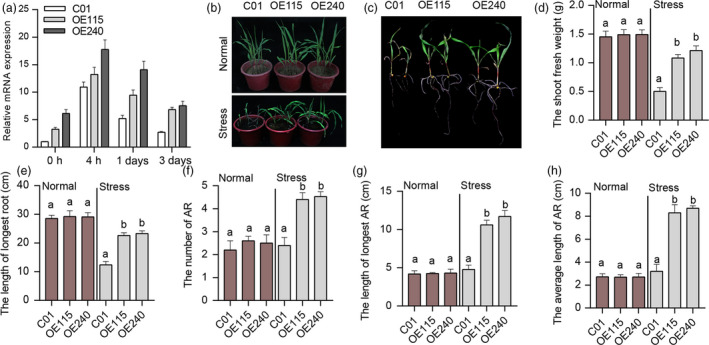
Phenotype of overexpression *ZmEREB180* maize lines under waterlogging stress. The overexpression *ZmEREB180* maize lines (OE115 and OE240) and C01 were subjected to waterlogging stress at the second‐leaf seedling stage, and the expression level of *ZmEREB180* was analysed. The phenotype was investigated after 16 days of waterlogging stress. (a) The *ZmEREB180* expression level of C01, OE115 and OE240 before (0 h) and after 4 h, 1 and 3 days of waterlogging stress. (b) The morphological phenotype of C01, OE115 and OE240 under Normal and after Stress condition. (c) The root and shoot characterizations of C01, OE115 and OE240 after Stress condition. (d–h) Phenotypic differences in terms of shoot fresh weight, length of longest root, number of ARs (adventitious roots), length of longest AR and average AR length of C01, OE115 and OE240 under Normal and Stress conditions, respectively. Data represent means ± SD of three independent replicates. C01 is a transgenic receptor, and OE115 and OE240 are two independent transgenic lines. The letters above the columns indicate a statistically significant difference (*P* < 0.05) for the C01 data compared to transgenic lines under Normal and Stress conditions, respectively. Normal, a controlled growth condition that was parallelly conducted; Stress, waterlogging treatment.

We also examined the waterlogging tolerance of transgenic maize transformed by *ZmUbi:ZmEREB180* under waterlogging stress. Two independent transgenic lines (OE115 and OE240) were analysed in the T_2_ generation. The transgenic lines overexpressed *ZmEREB180* by fourfold to sevenfold compared to the transformation receptor (C01) and that waterlogging stress enhanced the expression until 3 days in transgenic lines but not in C01 (Figure 3a). We subsequently investigated dynamic growth under normal and waterlogged conditions in parallel over 6 days. There were no significant differences in seedling height under normal conditions (Figure S6a), but seedling height in the transgenic lines was significantly higher than in C01 from the fourth day (Figure S6b), and SPAD values of the 1st leaf of transgenic lines were significantly higher than in C01 from the third day under waterlogging conditions (Figure S6d). In addition, there were no significant changes in transgenic lines before waterlogging treatment, measured in terms of shoot fresh weight, root fresh weight and root length (Figure S6c,f,g). However, measured traits increased in these lines after 6 days of waterlogging stress, and significant alterations were observed in transgenic lines compared to C01. Moreover, there was significantly less leaf injury in transgenic lines compared to C01 (Figure S6e). These results suggest that overexpression of *ZmEREB180* does not significantly influence the growth of maize seedlings under normal conditions, but transgenic plants strongly maintain growth ability under waterlogged conditions.

**Figure 4:**
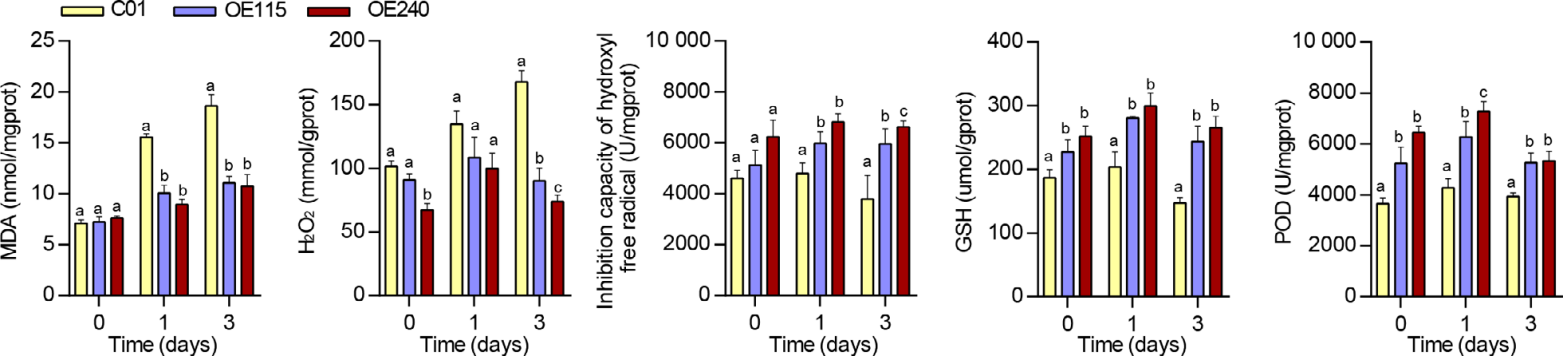
Physiological characteristics of overexpression *ZmEREB180* maize lines. C01 is a transgenic receptor, and OE115 and OE240 are two independent transgenic line. Data represent means ± SD of three independent replicates. MDA, malondialdehyde; POD, peroxidase; GSH, glutathione, H_2_O_2_, hydrogen peroxide. The letters above the columns indicate a statistically significant difference (*P* < 0.05) for the C01 data compared to transgenic lines at different time intervals of waterlogging stress.

We further observed enhanced tolerance under long‐term waterlogging stress in transgenic lines, which generated at least two complete green leaves (Figure 3b,c). The transgenic lines had significantly higher shoot fresh weight and length of the longest root compared with C01 (Figure 3d,e). Furthermore, the transgenic lines had well‐developed ARs compared to C01 (Figure S7), measured in terms of number of ARs, maximum AR length and average AR length (Figure 3f–h). Nevertheless, there is no obvious difference of the seedling characterizations that parallelly measured between transgenic lines and C01 under normal condition (Figure 3). Since oxidative stress, which affects plant development, was imposed on roots under waterlogged conditions, five physiological parameters that characterize oxidation state were also measured in OE115, OE240 and C01 seedling roots. The MDA content was not significantly different between transgenic maize lines and C01 under normal conditions, but C01 exhibited significantly higher MDA after 1 and 3 days of waterlogging stress (Figure 4). The content of H_2_O_2_ was significantly higher in C01 than in transgenic lines after 3 days stress (Figure 4), and inhibition capacity of hydroxyl free radical, representing the capacity of scavenging hydroxyl free radical, was significantly higher in transgenic lines than in C01 after 1 and 3 days of stress. The POD activity and GSH contents were significantly higher in transgenic lines than in C01 whether the stress was imposed or not. These results suggest that overexpressing *ZmEREB180* could increase antioxidants to alleviate oxidative damage and indicate that increased expression of *ZmEREB180* may promote AR development and enhance antioxidant ability as mechanisms for adapting to waterlogging stress.

### Multidimensional responses of nucleus‐localized *ZmEREB180* to waterlogging

**Figure 5:**
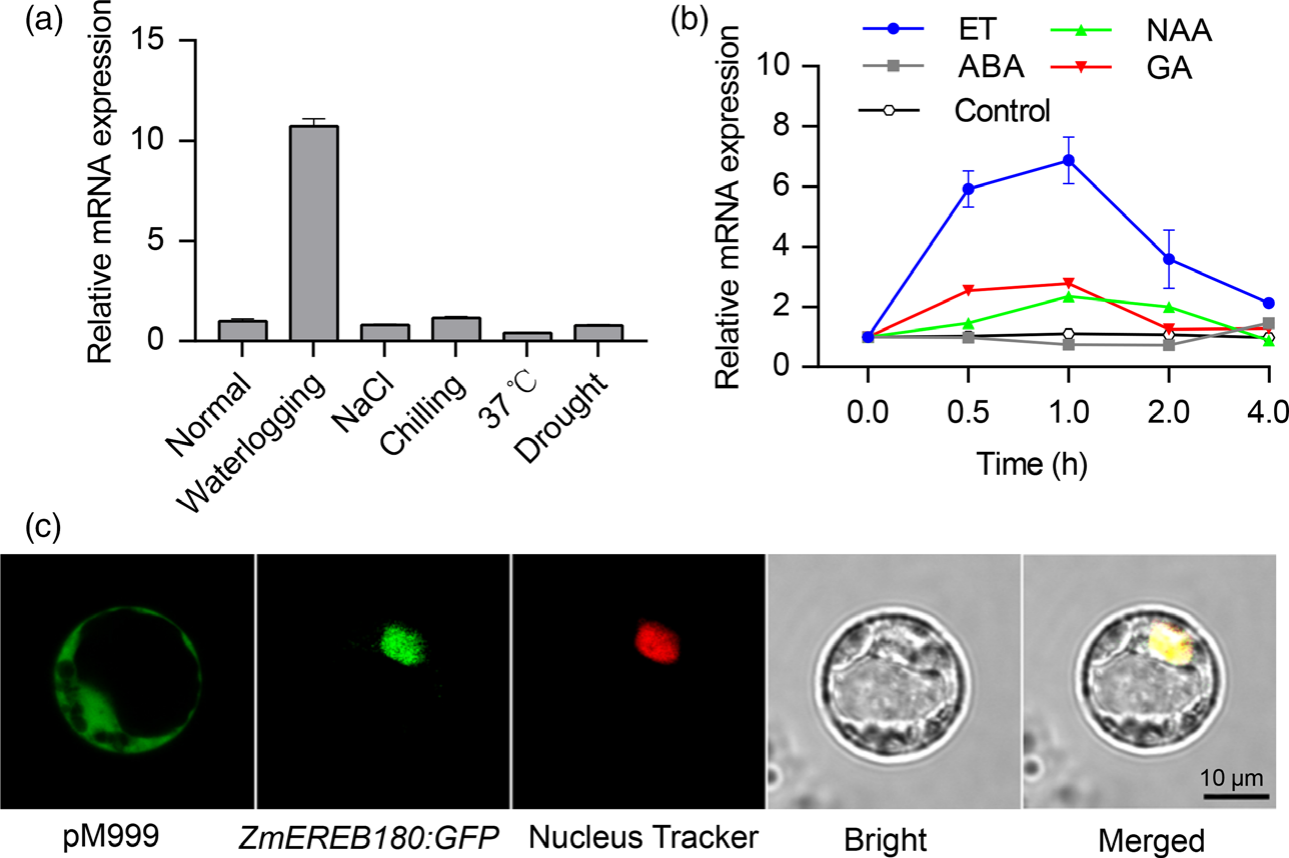
Response of *ZmEREB180* to abiotic stress and hormone‐treated seedling analysis of *ZmEREB180* subcellular localization. B73 seedlings were subjected to abiotic stress and hormones, and root samples of treated seedlings were used to quantify expression levels of *ZmEREB180*. (a) Expression level of *ZmEREB180* (4 h) under waterlogging, NaCl, chilling, 37 °C and drought stress. (b) Expression profiling of *ZmEREB180* after treatment with ET, NAA, ABA and GA. Control indicates that the root samples were collected under water conditions. (c) Analysis of subcellular localization of the ZmEREB180‐GFP fusion protein. *ZmEREB180‐GFP* fusion was transiently expressed in maize protoplasts, and GFP signals were detected by confocal fluorescent microscopy. Control maize protoplasts express pM999:GFP. ET, ethylene; NAA, 1‐naphthylacetic acid; ABA, abscisic acid; GA, gibberellin.

Given that *ZmEREB180* is an ethylene (ET) response factor gene and its orthologs in *Arabidopsis* (*HRE1* and *HRE2*) specifically respond to hypoxia (Licausi *et al*., 2010), we analysed the mRNA abundance of *ZmEREB180* under different stress and hormone treatments in B73 seedling roots. *ZmEREB180* specifically responded to waterlogging, while the NaCl and chilling treatments did not affect mRNA abundance (Figure 5a). *ZmEREB180* was markedly induced by ET and GA and NAA treatments but not by ABA (Figure 5b). Moreover, the GFP fluorescence of *ZmEREB180*‐*GFP* expressed in maize protoplasts colocalized with the Nucleus‐Tracker Red‐labelled nuclei (Figure 5c), indicating that ZmEREB180 targets to the nucleus. These results collectively demonstrate that ZmEREB180 is a nucleus‐localized ethylene response factor and specifically responds to waterlogging.

**Figure 6:**
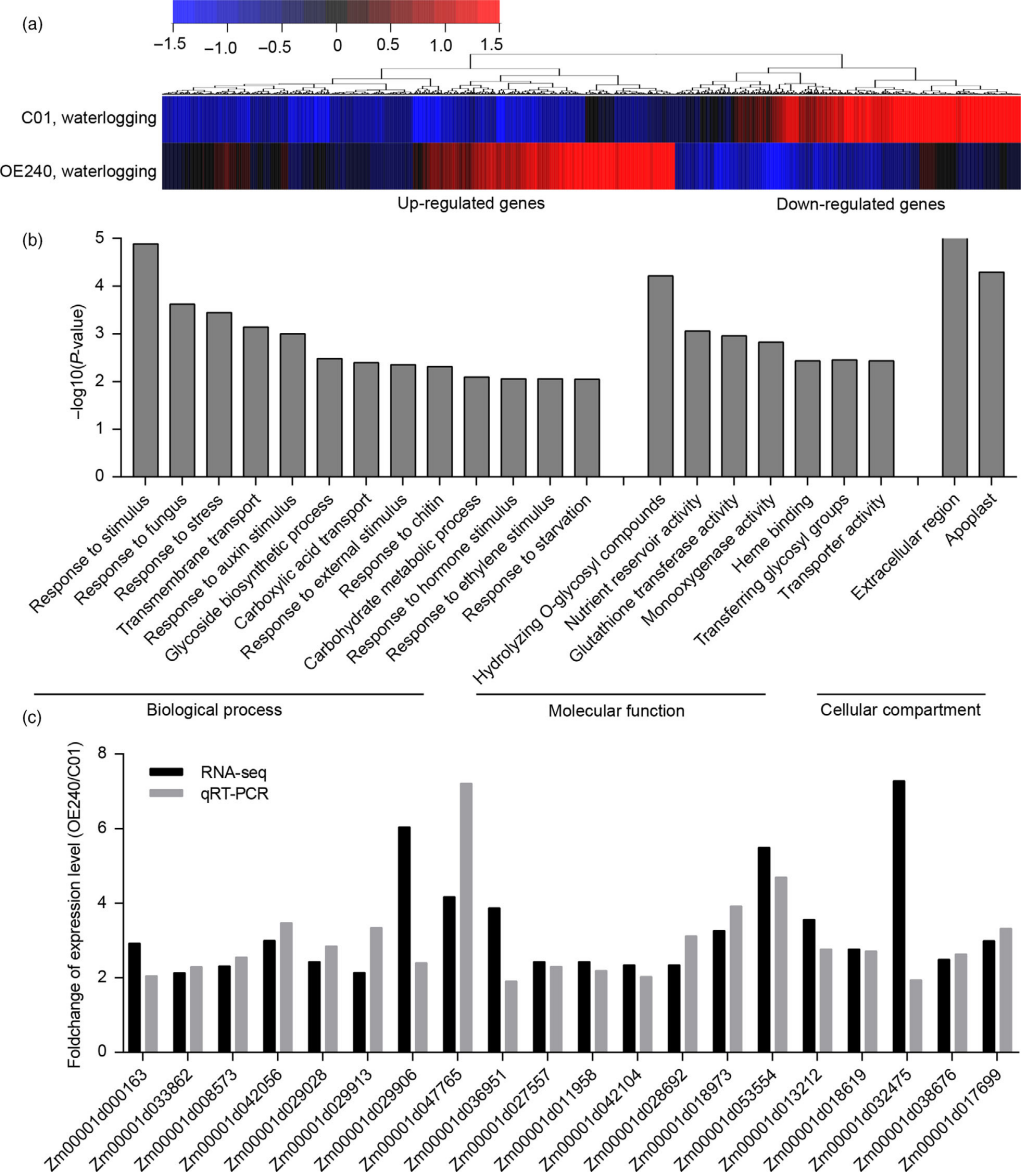
Transcriptomic analysis of the *ZmEREB180*‐overexpressing maize line under waterlogging stress. (a) Hierarchical clustering of differentially expressed genes in OE240 relative to C01 plants. (b) Significantly enriched GO in terms of up‐regulated genes in OE240. (c) qRT–PCR verification of up‐regulated expression genes in OE240 under waterlogging stress. C01 is a transgenic receptor, and OE240 is an independent transgenic line.

To investigate the regulatory network involving *ZmEREB180*, transcriptomes of the transgenic line OE240 and C01 plants under normal and waterlogged conditions were compared. A total of 1423 and 730 genes (adjust *P *<* *0.01, fold change >2 or <0.5) were up‐regulated and down‐regulated in OE240 compared to C01 under normal conditions, respectively (Figure S8a, Tables S4 and S5). Gene Ontology (GO) analysis revealed that the up‐regulated list was mainly enriched for response to stress, including fungus, wounding, chitin, hypoxia and hormone stimuli such as ethylene, auxin, jasmonic acids and hormone‐mediated signalling (Figure S8b). The most enriched category was regulation of transcription, which supports the conclusion that ZmEREB180 is a transcription factor. At the level of molecular function, protein serine/threonine kinase activity, transcription factor activity and calcium/haem binding were the most enriched categories. These results indicate that *ZmEREB180* is involved in a stress‐related pathway. Under waterlogging stress, 662 and 431 genes were twofold up‐regulated and down‐regulated in OE240 relative to C01, respectively (Figure 6a, Tables S6 and S7). GO analysis also showed that up‐regulated genes were most enriched for stimulus‐related pathways, including response to fungus, external stimuli, chitin, hormone stimuli and starvation (Figure 6b). Auxin and ET response, two endogenous hormone stimulus pathways, were enriched among the up‐regulated gene list; these are the two most important modulators of AR formation (Bellini *et al*., 2014). Programmed cell death (PCD) of the epidermal layers adjacent to AR primordia is a key phase before AR emergence (Atkinson *et al*., 2014). Among the 662 up‐regulated genes, many genes involved in PCD were identified, including those related to cell wall hydrolysis (three xyloglucan endotransglucosylase), cell wall loosening (six expansions) and Ca^2+^ signalling related (six calmodulin‐related genes). Moreover, eight glutathione transferase genes that are critical for H_2_O_2_ detoxification (Dixon *et al*., 2010) were significantly up‐regulated in OE240 under waterlogging stress, which was reflected in the enrichment in glutathione transferase activity at the GO molecular function level (Figure 6b). The increased expression of these genes was further validated by qRT–PCR analysis (Figure 6c, Table S2). These genes may be directly or indirectly involved in ZmEREB180‐mediated signalling to coordinate development and defence under waterlogging stress.

## Discussion

There are 98 members of the plant‐specific ERF transcription factor family in maize (Liu *et al*., 2013) and over 100 members in rice and *Arabidopsis*, all of which share an AP2 DNA‐binding domain (Nakano *et al*., 2006). ERFVIIs, a subgroup of the ERF family characterized by two conserved amino acid signatures within the AP2 domain (Magnani *et al*., 2004), are involved in a wide range of plant growth, development and stress responses (Gibbs *et al*., 2015). Fifteen rice ERFVIIs have been identified, with only *SUB1C* (OsERF73) classified as a subgroup b ERFVII (ERFVIIb); members of this subgroup lack the conserved N‐terminal region found in the ERFVIIa subgroup (Singh *et al*., 2010; Bailey‐Serres *et al*., 2012). *Arabidopsis* encodes five ERFVIIs that belong to ERFVIIa (Nakano *et al*., 2006). In this study, 19 *ZmERFVII* genes were identified in the maize genome (Figure S1), a number higher than that found in rice and *Arabidopsis*. We consider maize to contain fewer than the 20 *ZmERFVIIs* reported by Liu *et al*. (2013) because *ZmEREB118* did not share the conserved residue at position 18 of the AP2 domain and was therefore removed. Seven of the 19 *ZmERFVIIs* belonged to subgroup b (Figure S2), in marked contrast to rice and *Arabidopsis* (Nakano *et al*., 2006; Singh *et al*., 2010), indicating possible functional divergence in maize since amino acid variations often directly affect protein function. However, most of the *ZmERFVIIs* share the conserved N‐terminal motif, suggesting that similar functions to ERFVIIs in rice and *Arabidopsis* may also exist.

Investigations of *Arabidopsis* and rice revealed that ERFVII family members play a vital role in response to flooding. Four out of five ERFVIIs in *Arabidopsis* (*HRE1*,* HRE2*,* RAP2.12* and *RAP2.2*) are involved in anoxia/hypoxia stress (Licausi *et al*., 2010; Hinz *et al*., 2010; Hess *et al*., 2011; Licausi *et al*., 2011) and three ERFVII members in rice (*SUB1A*,* SK1* and *SK2*) confer submergence tolerance (Hattori *et al*., 2009; Xu *et al*., 2006). Given that homologous genes that evolved from the same ancestor can have a similar function, we hypothesized that ERFVIIs in maize may also respond to waterlogging, which is frequently encountered in the maize life cycle. Candidate gene association was thus conducted to identify associations between variations in *ZmERFVII* genes and SR phenotype in our association panel (Table 1). Some possible signals were identified, implying that these *ZmERFVIIs* are potentially involved in regulating waterlogging tolerance. *ZmEREB180* was associated with waterlogging tolerance in multiple environments and as such is a key candidate for importance in waterlogging tolerance (Table 1). *ZmEREB180* has a conserved N‐terminal motif (Figure S2), and ectopic expression *ZmEREB180* in *Arabidopsis* increased survival after submergence treatment (Figure S4), indicating that *ZmEREB180* functions similarly to *Arabidopsis* ERFVIIs such as *HRE1* (Licausi *et al*., 2010) and *RAP2.2* (Hess *et al*., 2011). Overexpression of *ZmEREB180* in maize further confirmed that transgenic lines exhibited increased survival ability under long‐term waterlogging stress (Figure 3). These results collectively demonstrate that *ZmEREB180* confers waterlogging tolerance at the maize seedling stage.

Molecular and physiological characterization of the key candidate genes involved in plant waterlogging tolerance is needed to understand the adaptation of plants to excess water conditions. However, very few loci associated with waterlogging stress have hitherto been cloned and used in breeding to enhance the waterlogging tolerance of maize; instead, most related research has involved mapping to wider genetic intervals along chromosomes (Mano and Omori, 2013; Mano *et al*., 2005; Qiu *et al*., 2007; Watanabe *et al*., 2017; Zaidi *et al*., 2015). Almost all of these studies were conducted using morphological‐ and biomass‐related traits, such as formation of aerenchyma and adventitious roots, root length, seedling height, fresh and dry weight of seedlings, and formation of radial oxygen loss barriers that indirectly reflect adaptive ability to waterlogging stress. In this study, the SR phenotype of inbred line seedlings was evaluated under long‐term stress (Figure S3), showing substantial differences in response to waterlogging. Recently, candidate gene association analysis combining the SR phenotype of drought tolerance in maize seedlings with natural variations in certain genes has successfully been conducted to identify favourable alleles of *ZmDERB2.7*,* ZmNAC111*,* ZmPP2C‐A10* and *ZmVPP1* for enhancing maize drought tolerance (Liu *et al*., 2013; Mao *et al*., 2015; Wang *et al*., 2016; Xiang *et al*., 2017). The same method was used in this study, and a favourable allele (Hap1) of *ZmEREB180* was detected, demonstrating that this method is also suitable for dissecting the genetic basis of waterlogging tolerance traits. Hap1 of *ZmEREB180* is considered to be favourable because (i) waterlogging‐tolerant maize inbred lines tend to exhibit Hap1, while waterlogging‐sensitive lines tend to exhibit Hap2 (Figure 1d); (ii) variants of Hap1 and Hap2 in *ZmEREB180* are highly associated with gene expression; that is, lines with Hap1 showed higher expression of *ZmEREB180* during waterlogging stress and tended to be waterlogging‐tolerant, whereas lines with Hap2 showed lower expression of *ZmEREB180* and tended to be waterlogging‐sensitive (Figure 2). Moreover, *ZmEREB180* is located within a genomic interval previously identified as associated with root length under waterlogging conditions (Osman *et al*., 2013). Therefore, InDel59 could be applied in breeding programmes to screen tolerant maize varieties to improve maize waterlogging tolerance.

As a simple gaseous phytohormone, ethylene plays a key role in plant responses to biotic and abiotic stresses (van Loon *et al*., 2006; Morgan and Drew, 1997) and is a major regulator of several flooding‐adaptive plant traits (Sasidharan and Voesenek, 2015). *SUB1A* and *SK1/SK2* confer opposite submergence‐adapted responses in rice and are induced by ethylene (Hattori *et al*., 2009; Xu *et al*., 2006), and *RAP2.2* in *Arabidopsis* regulates the ability of plants to resist hypoxia stress via an ethylene‐controlled signal transduction pathway (Hinz *et al*., 2010). In this study, *ZmEREB180* was also ethylene‐induced and specifically responded to waterlogging stress (Figure 5), and overexpressing *ZmEREB180* enhanced the formation of adventitious roots under waterlogged conditions (Figure 3). Adventitious roots facilitate gas transport and water and nutrient uptake during flooding to ensure plant survival (Sauter, 2013; Steffens and Rasmussen, 2016), which is a key response of many species such as rice (Lorbiecke and Sauter, 1999), maize (Zhai *et al*., 2013), *Rumex* spp. (Visser *et al*., 1996), tamarack (*Larix laricina*; Calvo‐Polanco *et al*., 2012) and *Eucalyptus* spp. (Argus *et al*., 2015). Ethylene is the major hormone that induces adventitious root growth in rice (Lorbiecke and Sauter, 1999) and tomato (Kim *et al*., 2008; Negi *et al*., 2010; Vidoz *et al*., 2010). Ethylene‐mediated development of adventitious roots in rice requires auxin signalling (Pacurar *et al*., 2014). These two hormones synergistically promote the growth of adventitious root primordia, and subsequent epidermal PCD above root primordial and adventitious root emergence are also regulated through ethylene (Atkinson *et al*., 2014; Steffens and Rasmussen, 2016). Transcriptome analysis on root samples of the *ZmEREB180*‐overexpressing transgenic line OE240 and maize inbred line C01 showed that the gene categories of auxin and ethylene signalling and genes involved in PCD were markedly enriched in OE240 in comparison with C01 under waterlogged conditions (Figure 6, Table S5), and they are critical for the formation of adventitious roots. This suggests that *ZmEREB180* regulates the formation of adventitious roots to enhance waterlogging tolerance.

Reactive oxygen species (ROS) are generated under flooding conditions, with the amount of ROS determined via either increased production of ROS or changes in antioxidant levels, which directly damage cells and cause lipid peroxidation (Bouranis *et al*., 2013; Steffens, 2014). ROS signals have also been implicated in mediating ethylene‐induced adventitious root growth through altering epidermal cell fate (Steffens *et al*., 2012). The genes that encode GST and POD were specifically enriched and up‐regulated in the transgenic line OE240 (Figure 6, Table S5), and the activity of POD and the content of GSH were significantly higher in OE240 than C01 whether waterlogging stress occurred or not (Figure 4). Primary roots were relatively more intact in transgenic lines than in C01 (Figure 3), which agrees with the lower MDA and H_2_O_2_ concentration and higher inhibition capacity of hydroxyl free radical, GSH content and the activity of POD in transgenic lines after waterlogging stress (Figure 4). Moreover, overexpressing *ZmEREB180* in *Arabidopsis* also demonstrated that transgenic lines had an enhanced recovery ability (Figure S4) and lower MDA and H_2_O_2_ content (Figure S5) after submergence treatment. These results indicate that *ZmEREB180* coordinates ROS homeostasis to mitigate oxidant damage in plants.

Based on these results, we proposed a model explaining how *ZmEREB180* enhances survival ability in waterlogging stress, although further direct molecular evidence will be needed. In our model, increased ethylene levels, stimulated by the onset of waterlogging, activate *ZmEREB180* expression. *ZmEREB180*, as an activator for up‐regulating the mRNA abundance of genes encoding endogenous hormones, subsequently promotes root primordial initiation through auxin and ethylene signalling and epidermal cell death through PCD. *ZmEREB180* also enhances the mRNA level of genes encoding antioxidants to regulate ROS status via changing antioxidant levels to sustain cell homeostasis, but whether *ZmEREB180* in maize is directly involved in epidermal PCD is unclear. Our results thus provide clues for understanding the molecular mechanisms underlying waterlogging stress responses and open the door for genetic improvements in breeding programmes.

## Experimental procedures

### Plant growth and waterlogging treatment

To investigate the survival rate of inbred lines in an association panel, seeds were planted in a temperature‐controlled greenhouse (~28 °C/22 °C day/night cycle) with 60% average humidity at Huazhong Agricultural University, Wuhan, China. A commercial growing mix (SunGro Horticulture, Agawam, MA) was used for the substrate, and 10 uniform seedlings of each line were planted in a plastic pot (20 cm in diameter and 30 cm deep) containing adequate substrate and sterile deionized water. Waterlogging protocols were similar to those described previously (Yu *et al*., 2018) and were applied at the second‐leaf stage by maintaining a 2‐ to 3‐cm water layer above the substrate. Three potted plant experiments were conducted using a randomized complete‐block design with three replicates. Root samples for gene expression analyses were collected directly before waterlogging treatment (control), after 4 h (short‐term) of stress and after 3 days (long‐term) of stress.

### Evaluating waterlogging tolerance in the maize association panel

A maize association panel consisting of 368 inbred lines (Li *et al*., 2013) was planted, and survival rates (SRs) were recorded, calculated as the ratio of the number of surviving plants to the total number of plants in each plot. The time point for recording the SR was determined by the performance of inbred line B73. When the SR of B73 decreased to 50%, the SR of the other inbred lines was assessed. The average value of three replicates per genotype was calculated to represent the SR phenotype, and the average SR value from three different environments was estimated using the BLUP via linear mixed models, in which genotype and environment were set as random effects.

### Association analysis of *ZmERFVII* genes

Association analysis of *ZmERFVIIs* was performed using the SR phenotypes from three experiments and BLUP data. Among 525 105 high‐quality SNPs with a minor allele frequency (MAF) of ≥5% in the association panel (Fu *et al*. 2013), 607 SNPs were located within the gene region of all 19 *ZmERFVIIs*. A cMLM was estimated in TASSEL v3.0 (Bradbury *et al*., 2007) to quantify significant associations as functions of population structure (Q matrix) and familial kinship (K matrix; Yu *et al*., 2005; Zhang *et al*., 2010). Two thresholds (*P *<* *0.01; *P *<* *0.001) were used for determination of significant trait–SNP associations.

### *ZmEREB180* gene sequence and association with waterlogging tolerance

For the purpose of *ZmEREB180* resequencing, three pairs of primers (Table S2) were synthesized to amplify the promoter (1.1 kb), 5′‐ and 3′‐UTR (untranslated region), and all introns and exons of the gene in 248 randomly selected inbred lines from the association panel, using the B73 genome sequence as a reference (B73 RefGen_v4, Jiao *et al*., 2017). All amplified sequences were aligned using MEGA v5 (Tamura *et al*., 2011). Nucleotide polymorphisms including SNPs and InDels were identified, and variants with MAF ≥5% were used for association analysis. Variants significantly associated with SR according to BLUP data were calculated again using the cMLM.

### *ZmEREB180* gene expression analysis

For analysis of *ZmEREB180* mRNA abundance in 100 maize inbred lines, root samples were separately collected from seedlings grown under normal (0 h), short‐term stress (4 h) and long‐term stress (3 days) treatments, and roots from five seedlings were pooled for RNA extraction. Total RNA from 300 samples was isolated using TRIZOL reagent (Invitrogen, Gaithersburg, MD) and treated with RNase‐free DNase (Invitrogen). The purified RNA was used to synthesize single‐stranded cDNA using recombinant M‐MLV reverse transcriptase (Invitrogen). Quantitative reverse‐transcription PCR (qRT–PCR) was performed using gene‐specific primers (Table S2) with 2× iTaq™ Universal SYBR Green Supermix (Bio‐Rad, Hercules, CA). *ZmActin1* (GRMZM2G126010) was employed as the internal control to normalize the expression data. Relative expression levels were calculated according to the 2^−ΔΔ*C*T^ (cycle threshold) method (Livak and Schmittgen 2001). PCR involved an initial denaturation step at 95 °C for 5 min, followed by 40 cycles at 95 °C for 15 s, 58 °C for 10 s and 72 °C for 20 s.

To analyse the expression regulation of *ZmEREB180* under stress and hormone treatment, waterlogging, sodium chloride (NaCl, 200 mm), chilling (10 °C), high temperature (37 °C) and drought stress were imposed on the second‐leaf stage B73 seedlings, and root samples were collected after 4 h of stress. For hormone treatment, the second‐leaf stage B73 seedlings were cultured in water with the following treatments: ethylene (ET, 100 μm), abscisic acid (ABA, 100 mm), gibberellin (GA, 100 mm) and 1‐naphthaleneacetic acid (NAA, a synthetic plant hormone in the auxin family, 100 mm; Sigma‐Aldrich, Shanghai, China), with covering used in the ET treatment to avoid volatilization. Root samples were collected at 0.5, 1, 2 and 4 h after hormone treatment, and the seedling roots with no additional chemical substance were also sampled as control. Extraction, purification and expression assays were conducted as described above.

For analysis of subcellular localization of *ZmEREB180*, the full‐length coding sequence of *ZmEREB180* in B73 seedlings was amplified and inserted into the pM999‐GFP vector to generate a GFP fusion construct (*ZmEREB180‐GFP*). The *ZmEREB180‐GFP* construct was placed under the cauliflower mosaic virus 35S promoter for constitutive expression. Maize mesophyll protoplasts were isolated from etiolated leaves, and the fusion construct was introduced into protoplasts with polyethylene glycol (PEG)/calcium‐mediated transformation (Ren *et al*., 2017; Yoo *et al*., 2007). Nucleus‐Tracker Red was used for nuclear labelling.

For transient expression assays in maize protoplasts, two different types of promoter (with 5ʹ‐UTR) sequence of inbred lines B73 and 835B that have the same sequence of promoter were cloned into vector pGreen0800 II (Zhang *et al*., 2016) to compare the differential expression of 5ʹ‐UTR, which was inserted in front of the mini35S promoter to drive the expression of the *LUC* reporter gene. The expression of the *LUC* reporter gene under mini35S promoter (Vector, Zhang *et al*., 2016) was set as background activity. These constructs were transformed into maize protoplasts, and Firefly and Renilla luciferase activities were quantified with a dual‐luciferase reporter assay according to the manufacturer's instructions (Promega, Madison, USA).

### *Arabidopsis* submergence tolerance assays

The *ZmEREB180* coding sequence in B73 inbred lines was amplified and inserted into pCAMBIA1300 under the *CaMV 35S* promoter using SmaI restriction sites. The recombinant plasmid was then transformed into *Agrobacterium tumefaciens* strain GV3101. *Arabidopsis thaliana* ecotype Col‐0 was transformed by *Agrobacterium*‐mediated transformation, and independent T_2_ transgenic lines were obtained using PCR and kanamycin‐based selection. Expression of *ZmEREB180* in transgenic plants was determined by qRT–PCR, with At5g08290 used as an internal control for normalization (Czechowski *et al*., 2005; Hinz *et al*., 2010). Two independent overexpression lines, OE5 and OE8, were selected based on the level of transgene expression and subjected to further analyses. For the submergence tolerance assays, 3‐week‐old plants were submerged under 13/11 day/night at 22 °C. Rosettes were submerged 5 cm below the water surface. Fresh weights of seedlings growing above the substrate were measured, and samples were photographed after 10 days of treatment following recovery for 7 days. Whole seedlings growing under 0 (normal), 4 and 8 h of stress were collected for qRT–PCR, and at least five seedlings were combined for RNA extraction. The expression of four anaerobic metabolism‐related genes (*AtADH1*,* AtPDC1*,* AtSUS1* and *AtSUS4*) was quantified in overexpression plants and Col‐0 (wild type, WT). Empirical data were obtained from three independent experiments. Gene‐specific primers used for qRT–PCR analysis are listed in Table S2.

### Generation and analysis of transgenic maize

The coding region of *ZmEREB180* was amplified from B73 and inserted into the pCAMBIA1300 vector using the Vazyme recombination kit under the control of the *Zmubi1* promoter. The constructed plasmid was then transformed into *Ag. tumefaciens* EHA105. The EHA105 strain with a recombinant plasmid was then used to deliver *Zmubi1:ZmEREB180* into the C01 maize inbred line. The China National Seed Group carried out the genetic transformation. Positive transgenic plants were determined in each generation by PCR analysis. Expression of *ZmEREB180* in transgenic plants was determined by qRT–PCR. Two independent T_2_ lines, OE115 and OE240, were selected for further analyses. Using a commercial growing mix as substrate (SunGro Horticulture), OE115, OE240 and C01 plants were planted in a light room under 13‐h light/11‐h dark, 25 °C day/22 °C night conditions. Waterlogging treatment was applied to plants at the second‐leaf stage. Phenotypes, namely, seedling height, root length, leaf injury (LI) and soil and plant analyser development (SPAD) values, of first leaves were measured before treatment (0 h) and 2, 4 and 6 days after the initiation of stress. After ~15 days, the SR of each line was recorded, as well as the longest root length, shoot fresh weight, number of adventitious roots (ARs), length of the longest AR and average AR length. Statistical analysis was based on data obtained from at least eight seedlings for each plant line with three independent experiments.

### Physiological assay

For analysis of physiological characteristics, root samples from no fewer than six maize seedlings for OE115, OE240 and C01 were collected at 0 h, 1 and 3 days after waterlogging stress at the second‐leaf stage, and no fewer than 10 seedlings for OE5, OE8 and WT of 3‐week‐old *Arabidopsis* plants were collected at 0 h, 1 and 2 days after submergence. Weighed samples were manually ground to a fine powder in liquid nitrogen and then homogenized in fourfold v/w 0.9% normal saline under ice‐cold conditions to form a 20% tissue homogenate. The homogenates were centrifuged (2500 ***g***, 10 min, 4 °C), and the supernatants were used for further analysis. Malondialdehyde (MDA), peroxidase (POD) and glutathione (GSH) measurements proceeded according to previous descriptions (Yu *et al*. 2015). Briefly, GSH concentrations were obtained using thiobis‐(2‐nitrobenzoic acid) for colour development and monitoring wavelengths at 420 nm, and the concentrations were expressed as micromoles per gram of protein; MDA measurements were based on the thiobarbituric acid condensation reaction, and the red product was monitored at 532 nm and expressed as nanomoles per milligram of protein. POD activity was assayed by assessing the oxidation of H_2_O_2_ at 420 nm; one unit was defined as the amount of enzyme that catalysed and generated 1 μg of product per 1 min by 1 mg of homogenate in the reaction system at 37 °C. The hydrogen peroxide (H_2_O_2_) concentrations and inhibition capability of hydroxyl free radical were measured using H_2_O_2_ assay kits and hydroxyl free radical assay kits, respectively (Nanjing Jiancheng Bioengineering Institute, Nanjing, China). Three replicates were performed for each assay.

### RNA‐seq analysis of transgenic maize

For maize RNA‐seq analysis, roots from five‐two‐leaf stage seedlings were collected from transgenic and C01 plants before and after 4 h of waterlogging stress. The samples from 0E115 and OE240 were pooled for total RNA isolation with three biological replicates. The 150‐bp paired‐end Illumina sequencing was conducted at the National Key Laboratory of Crop Genetic Improvement (Huazhong Agricultural University, Wuhan, China) using HiSeq2500 (Illumina Inc., San Diego, CA). An average of six gigabases of raw data were generated for each sample. A bioinformatic analysis was carried out as previously described (Du *et al*., 2017). High‐quality clean reads were aligned to the maize reference genome (B73 RefGen_v4, Jiao *et al*., 2017). GO enrichment analysis was performed using agriGO v2.0 (Tian *et al*., 2017). All RNA samples for transcriptome sequencing were also used to validate the mRNA abundance of the differential expression genes (Table S3).

## Conflict of interest

The authors declare that they have no conflict of interest.

## Supporting information


**Figure S1** Position of 19 ZmERFVIIs genes on the maize chromosome.
**Figure S2** Characterization of gene structure and putative conserved motif of ZmERF‐VIIs.
**Figure S3** Phenotypic distribution of survival rate (SR) in the association panel.
**Figure S4** Phenotype of ectopic expression of *ZmEREB180* in *Arabidopsis* under waterlogging stress.
**Figure S5** Physiological characteristics of overexpression *ZmEREB180 Arabidopsis* lines.
**Figure S6** Dynamic phenotypes of overexpressing *ZmEREB180* maize lines under waterlogging stress.
**Figure S7** Characteristics of adventitious roots of overexpressing *ZmEREB180* maize lines under waterlogging stress.
**Figure S8** Transcriptomic analysis of overexpressing *ZmEREB180* maize lines under normal conditions.
**Table S1** Gene identification information for 19 group VII EREB genes in versions 3 and 4 of the B73 reference genome.
**Table S2** Primers used for resequencing and expression analysis of ZmEREB180.
**Table S3** qRT‐PCR primers used for up‐regulation gene validation under waterlogging stress in RNA‐seq analysis.

**Table S4** Up‐regulated genes in OE240 seedling roots compared to C01 under normal conditions.
**Table S5** Down‐regulated genes in OE240 seedling roots compared to C01 under normal conditions.
**Table S6** Up‐regulated genes in OE240 seedling roots compared to C01 under waterlogging stress.
**Table S7** Down‐regulated genes in OE240 seedling roots compared to C01 under waterlogging stress.
