# Supplementary material for: A group VII ethylene response factor gene, ZmEREB180, coordinates waterlogging tolerance in maize seedlings
Source: Plant Biotechnol J. 2019 May 14;17(12):2286–98. doi: 10.1111/pbi.13140 (PMC6835127; doi:10.1111/pbi.13140)
Supplement: Supplementary file 1 — Figure S1 Position of 19 ZmERFVIIs genes on the maize chromosome. Figure S2 Characterization of gene structure and putative conserved motif of ZmERF‐VIIs. Figure S3 Phenotypic distribution of survival rate (SR) in the association panel. Figure S4 Phenotype of ectopic expression of ZmEREB180 in Arabidopsis under waterlogging stress. Figure S5 Physiological characteristics of overexpression ZmEREB180 Arabidopsis lines. Figure S6 Dynamic phenotypes of overexpressing ZmEREB180 maize lines under waterlogging stress. Figure S7 Characteristics of adventitious roots of overexpressing ZmEREB180 maize lines under waterlogging stress. Figure S8 Transcriptomic analysis of overexpressing ZmEREB180 maize lines under normal conditions. Table S1 Gene identification information for 19 group VII EREB genes in versions 3 and 4 of the B73 reference genome. Table S2 Primers used for resequencing and expression analysis of ZmEREB180. Table S3 qRT‐PCR primers used for up‐regulation gene validation under waterlogging stress in RNA‐seq analysis. [file PBI-17-2286-s001.docx]

## *Plant Biotechnology Journal* Supporting Information

Article title: A group Ⅶ ethylene response factor gene, *ZmEREB180*, coordinates waterlogging tolerance in maize seedlings

Authors: Feng Yu, Kun Liang, Tian Fang, Hailiang Zhao, Xuesong Han, Manjun Cai and Fazhan Qiu

The following Supporting Information is available for this article:

**Table S1. Gene identification information for 19 group VII EREB genes in versions 3 and 4 of the B73 reference genome.**

| Gene_code | chr | Gene_ID (V3) | Gene_ID (V4) | position_start | position_end |
| --- | --- | --- | --- | --- | --- |
| ZmEREB179 | 1 | GRMZM2G129674 | Zm00001d027924 | 17775779 | 17777344 |
| ZmEREB180 | 1 | GRMZM2G018984 | Zm00001d027925 | 17807124 | 17808643 |
| ZmEREB181 | 1 | AC206951.3_FG016 | Zm00001d027928 | 17833120 | 17833935 |
| ZmEREB182 | 1 | AC206951.3_FG017 | Zm00001d027929 | 17839868 | 17840788 |
| ZmEREB172 | 1 | GRMZM2G369472 | Zm00001d031796 | 202103550 | 202104359 |
| ZmEREB167 | 1 | GRMZM2G050851 | Zm00001d032095 | 212364322 | 212366865 |
| ZmEREB211 | 2 | GRMZM2G138396 | Zm00001d005892 | 191897839 | 191898630 |
| ZmEREB202 | 2 | GRMZM2G148333 | Zm00001d005798 | 189023745 | 189025630 |
| ZmEREB210 | 2 | GRMZM2G125460 | Zm00001d007033 | 220493755 | 220494609 |
| ZmEREB193 | 3 | GRMZM2G169382 | Zm00001d040651 | 56282555 | 56283595 |
| ZmEREB14 | 4 | GRMZM2G018398 | Zm00001d052087 | 179070181 | 179072822 |
| ZmEREB7 | 4 | GRMZM2G173771 | Zm00001d052167 | 181560436 | 181561128 |
| ZmEREB90 | 5 | GRMZM2G110333 | Zm00001d018305 | 218213866 | 218216075 |
| ZmEREB139 | 5 | GRMZM2G103085 | Zm00001d018158 | 215716411 | 215717121 |
| ZmEREB69 | 6 | GRMZM2G085964 | Zm00001d037941 | 142909977 | 142911255 |
| ZmEREB200 | 7 | GRMZM2G025062 | Zm00001d022461 | 178147095 | 178148306 |
| ZmEREB116 | 7 | GRMZM2G131281 | Zm00001d021089 | 142530982 | 142531634 |
| ZmEREB102 | 7 | GRMZM2G052667 | Zm00001d020595 | 124130947 | 124133914 |
| ZmEREB160 | 9 | GRMZM2G171179 | Zm00001d045044 | 10942420 | 10945088 |

**Table S2. Primers used for re-sequencing and expression analysis of *ZmEREB180*.**

| **The primer for re-sequencing of *ZmEREB180*.** | | |
| --- | --- | --- |
| Primer | Forward/Reverse | Sequence of primers |
| Primer1 | Forward | CAGCAACACGAACAACACGA |
|  | Reverse | GTCCGTTTAGCACGACTCCA |
| Primer2 | Forward | CCAACGGCGTACAAATCGAG |
|  | Reverse | AAGTTGACCTTGGCCTTGCT |
| Primer3 | Forward | GCCCGTCTTGTGTATAGCCC |
|  | Reverse | GCATTTGGATCGGAACGCTT |
|  |  |  |
| **qPCR primers used for *ZmEREB180* expression .** | | |
| Gene | Forward/Reverse | Sequence of primers |
| *ZmEREB180* | Forward | AGAGGAAGGAAGGGATCGC |
|  | Reverse | GAGTCTTCGTCGCATCTCG |
| *ZmActin1* | Forward | TACGAGATGCCTGATGGTCAGGTCA |
|  | Reverse | TGGAGTTGTACGTGGCCTCATGGAC |
|  |  |  |
| **qPCR primers used in transgenic *Arabidopsis* plants.** | | |
| Gene | Forward/Reverse | Sequence of primers |
| At5g08290 (control) | Forward | TTGAGACTGTCTACCGTGGTG |
|  | Reverse | CAAACTAGATAGTGTTGGGAAGCTC |
| *AtSUS1* | Forward | TTCAAAGCAATGCCACAGAG |
|  | Reverse | AGTGGTTCCGGTGTTTGAAG |
| *AtSUS4* | Forward | TGGAAACATGTCTCGAACCTT |
|  | Reverse | CTCTTCATGAGCAAGAGGAACA |
| *AtPDC1* | Forward | TGATGCTTCAGGCTATGCTTT |
|  | Reverse | GTTGAAGATTGGACCTGCAAA |
| *AtADH1* | Forward | TTGCTCCACCGCAGAAACAC |
|  | Reverse | CCAACACTCTCAACAATCCCTCC |
| **Primers used for detecting the significant loci in the 5ʹ-UTR of *ZmEREB180*** | | |
| Gene | Forward/Reverse | Sequence of primers |
| InDel59 | Forward | GCAAAACTAAGACTTTCTCTAGCA |
|  | Reverse | TGTGCCCTGTGTATTTTTCGACA |

**Table S3. qRT-PCR primers used for up-regulation gene validation under waterlogging stress in RNA-seq analysis.**

| Gene | Primer sequence (F/R) | Annotation |
| --- | --- | --- |
| Zm00001d047765 | AGCTGCTCGGATACACTGAC / GGCGTCTTCTATTTTGTCCA | Glutathione S-transferase L2 chloroplastic |
| Zm00001d036951 | GTACTCGGAGCAGGAGCTG /  GAGGTACTCGAGGATGACGA | Glutathione transferase19 |
| Zm00001d027557 | CTCTCAGAACTCGGGAAAAA /GGACGATGATCAGAGACTCG | Glutathione transferase31 |
| Zm00001d011958 | GAGGGAGTACCCAAACCTGT / GTAGTCGATGTTGGGTCCAG | Glutathione S-transferase family protein |
| Zm00001d042104 | GGGACTACAGCTCCAGTGAA / GCCTTGGCAAATGTCTTGTA | Glutathione transferase7 |
| Zm00001d028692 | TCCCTATGAACATGGCAGAT /  GTCGGTGATGGTAAGTTTGG | Glutathione S-transferase L2 chloroplastic |
| Zm00001d053554 | CTTCGACAACGCCTACTACC /  ACCCTACAGTTTGCCCTGAT | Peroxidase 52 |
| Zm00001d013212 | AGCTGCTCTACACCGACAAC / CAGTTGAACACGTCGCACT | Peroxidase 16 |
| Zm00001d018619 | ACCACAATGGAGAGATCAGG /CACAGCTAGAGCGAGTGACA | Peroxidase 1 |
| Zm00001d000163 | AGATCGAGGTGCTGAGCAAT / GGCTGGAACTTCTGCTTGAC | 1-aminocyclopropane-1-carboxylate oxidase |
| Zm00001d033862 | AGGTACATCGCGGAGAACAC /CCTTCTTCCACAGCTCCATC | 1-aminocyclopropane-1-carboxylate synthase6 |
| Zm00001d038676 | GTGGGTCGTCACCAAGAAGT /GTCAGCAGGAGGAAGGACAG | Probable xyloglucan glycosyltransferase 12 |
| Zm00001d017699 | TCGTCGCCTTCTACCTGTCT /  CCCAGAGGATGGAGTAGCTG | Probable xyloglucan endotransglucosylase |
| Zm00001d029906 | GGCTCCTGCTACCAGATACG /  CCCTCTTGAACTGGATGTCG | Beta-expansin 7 |
| Zm00001d029913 | CAGTACCCGTTCTCGTCCAT /  TACTTGGCCACCGGGTAGTA | Expansin-B4 |
| Zm00001d032475 | CTCAAGCACCCGCTCTTC /  GTCGAGGTCGACGAGGTG | SAUR56-auxin-responsive SAUR family member |
| Zm00001d018973 | AGGTCGAGCAGAAGCAGAAG /CGGAGAAGGAGTTGGTGAAG | IAA24-auxin-responsive Aux/IAA family member |
| Zm00001d008573 | AGATGCAGAGGGTGTTCTCG /  GTGTCCAGCTCGTCCATCAT | Calmodulin protein |
| Zm00001d042056 | GGATGTACTCCTCCGACGAC /  GCCCATCATGACCTTGAACT | Calcium-binding protein CML38 |
| Zm00001d029028 | TGTGACCGAGGATGACGAC /  GATCCCCTGCATCATGTTCT | Calcium-binding protein CML42 |


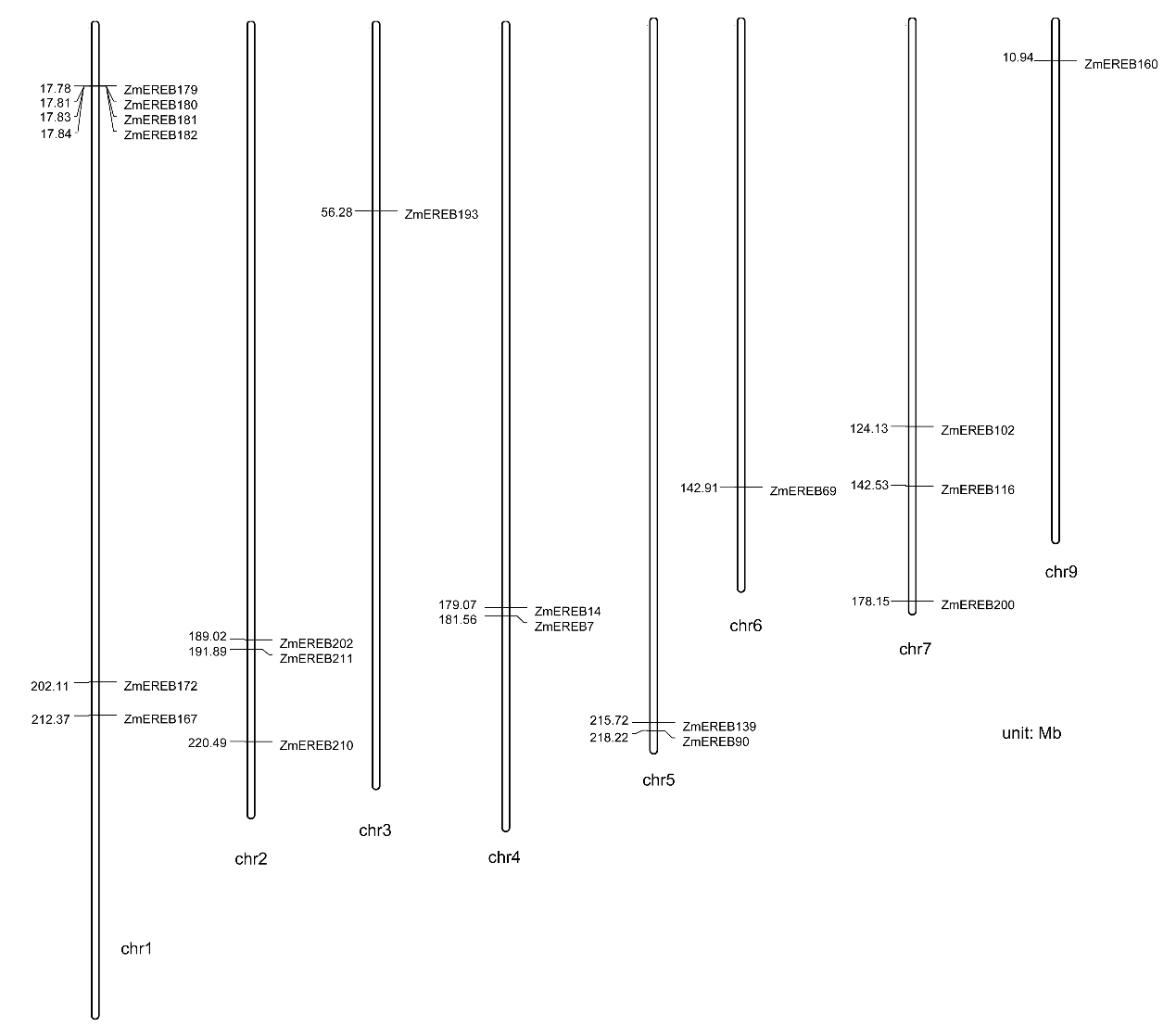


**Fig. S1. Position of 19 ZmERFVIIs genes on the maize chromosome.** The physical position of each gene is according to B73 reference genome V4.


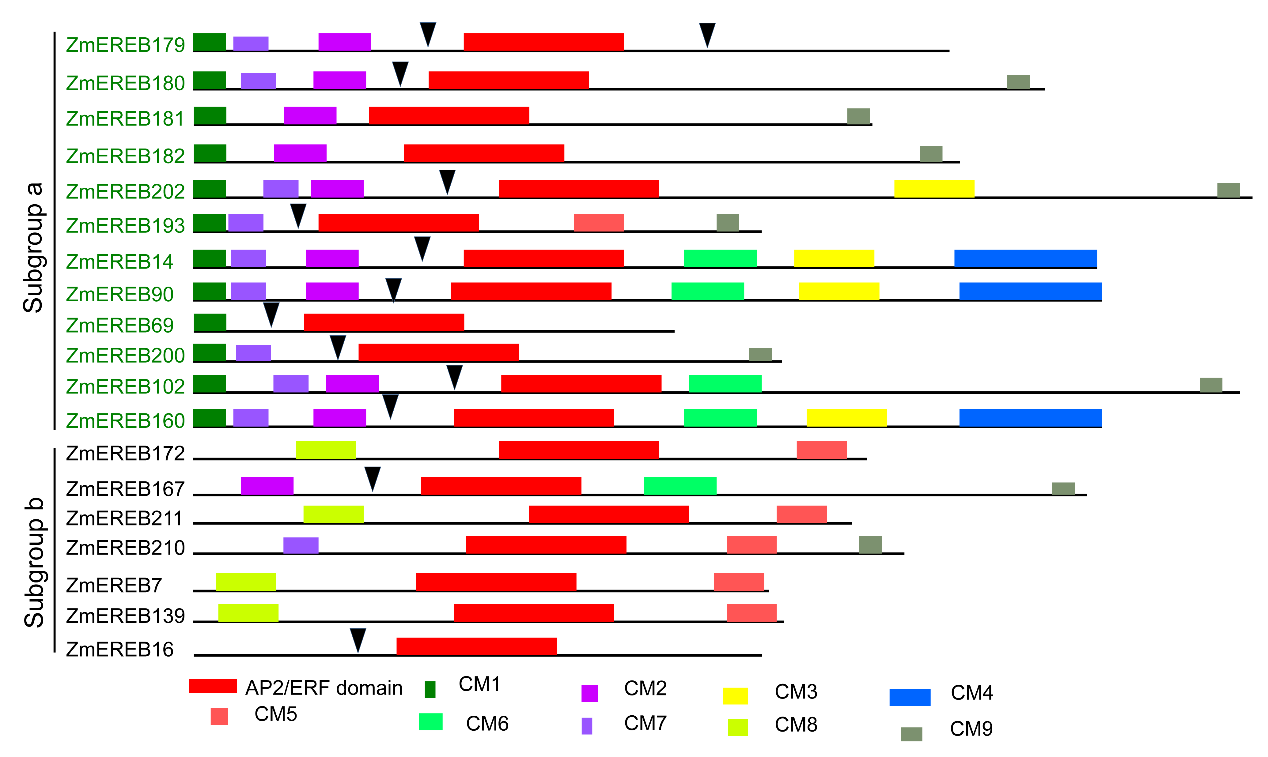


**Fig. S2. Characterization of gene structure and putative conserved motif of *ZmERF-VIIs*.** Gene structure was displayed by GSDS2.0 software. Putative conserved motifs shared by *ZmERFVIIs* proteins were mined in MEME program. Fifteen motifs are indicated by different colored boxes. Black inverted triangle indicates the position of intron.


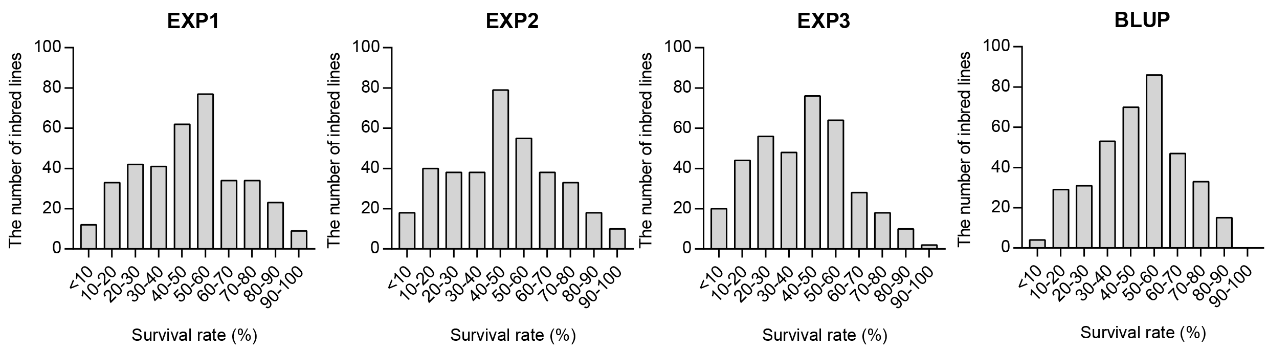


**Fig. S3. Phenotypic distribution of survival rate (SR) in the association panel.** EXP1, EXP2, EXP3 and BLUP represented SR phenotypes from environment1, environment2, environment3 and BLUP data.


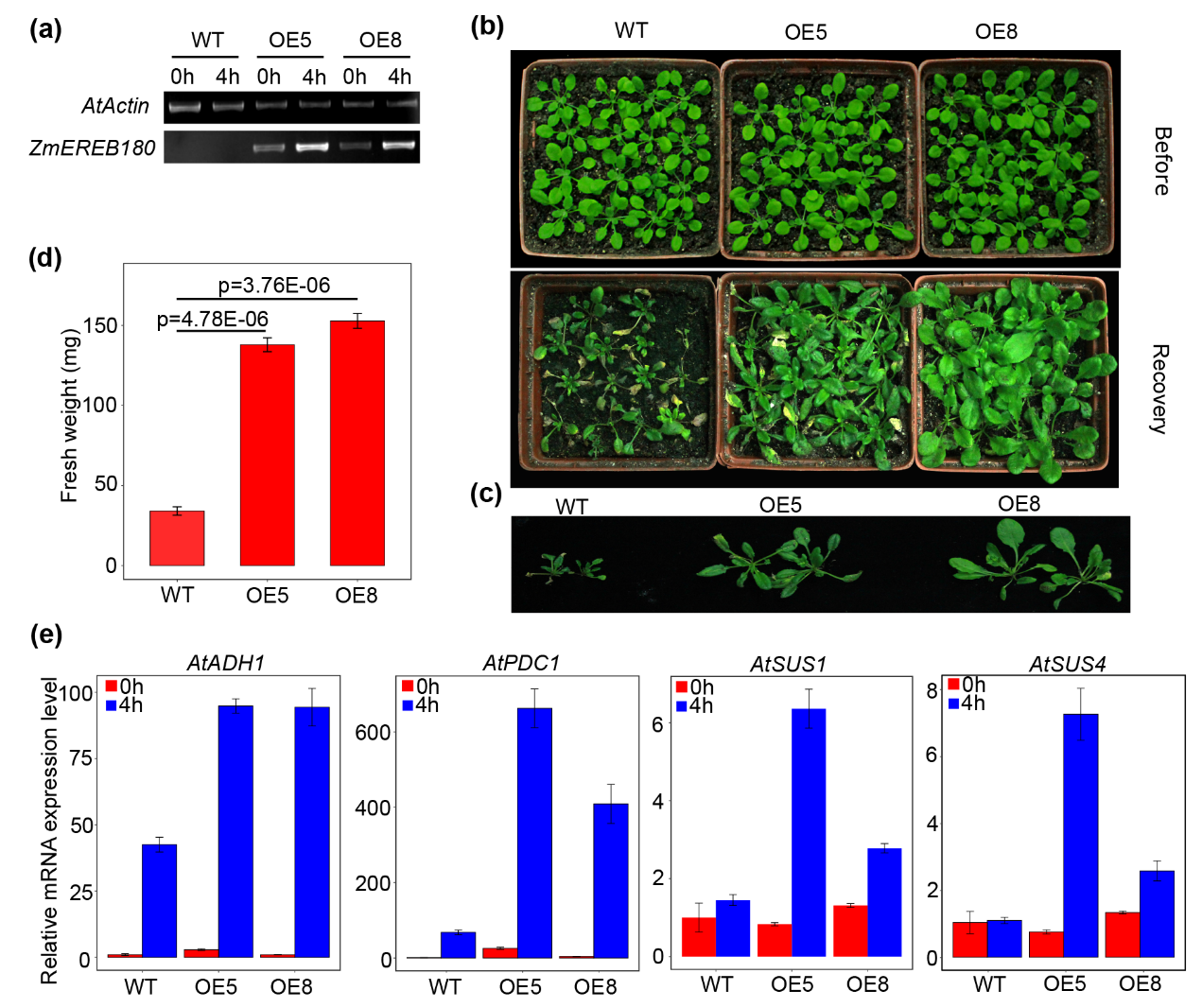


**Fig. S4. Phenotype of ectopic expression of *ZmEREB180* in *Arabidopsis* under waterlogging stress.** (a) The expression level of *ZmEREB180* in the overexpression lines (OE5 and OE8) of *Arabidopsis* and wild type (WT) before and after 4h submergence stress. (b) Comparative analysis of phenotype of OE5, OE8 and WT after submergence stress. (c) The morphological difference in aboveground seedlings of OE5, OE8 and WT after submergence stress. (d) The fresh weight of aboveground seedlings of OE5, OE8 and WT after submergence stress. (e) The expression level of four anaerobic marker genes in OE5, OE8 and WT. Three-week seedlings were used to investigate the expression level of *ZmEREB180* and phenotype after submergence stress. The phenotype was observed after 10 d submergence stress and 7 d subsequent recovery. OE5 and OE8 are two independent transgenic lines, WT is wild type of Arabidopsis.

**
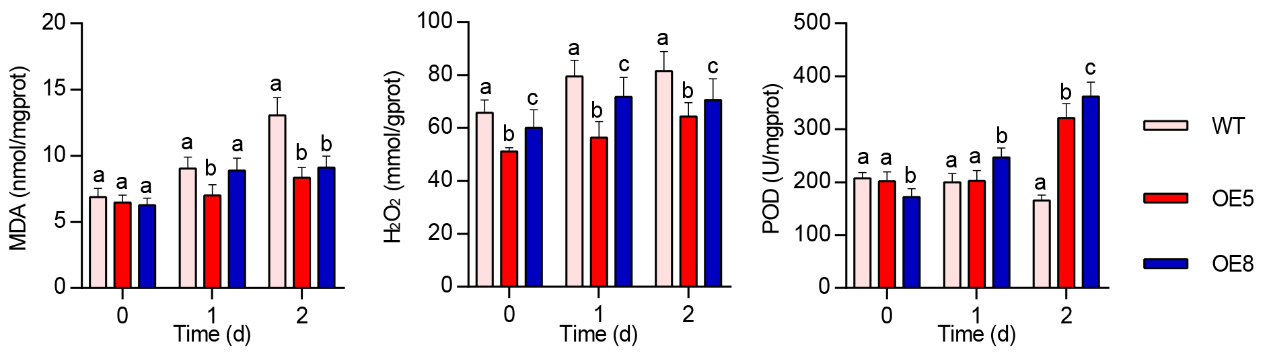
**

**Fig. S5. Physiological characteristics of overexpression *ZmEREB180* *Arabidopsis* lines.** Three-week plants were subjected to submergence stress. OE5 and OE8 are two independent transgenic lines, WT is wild type of Arabidopsis. MDA, Malondialdehyde; POD, peroxidase; H_2_O_2_, hydrogen peroxide. Data represent means ± SD of three independent replicates. The letters above column indicates a statistically significant difference for the data of WT compared to transgenic lines at different time intervals of submergence stress.


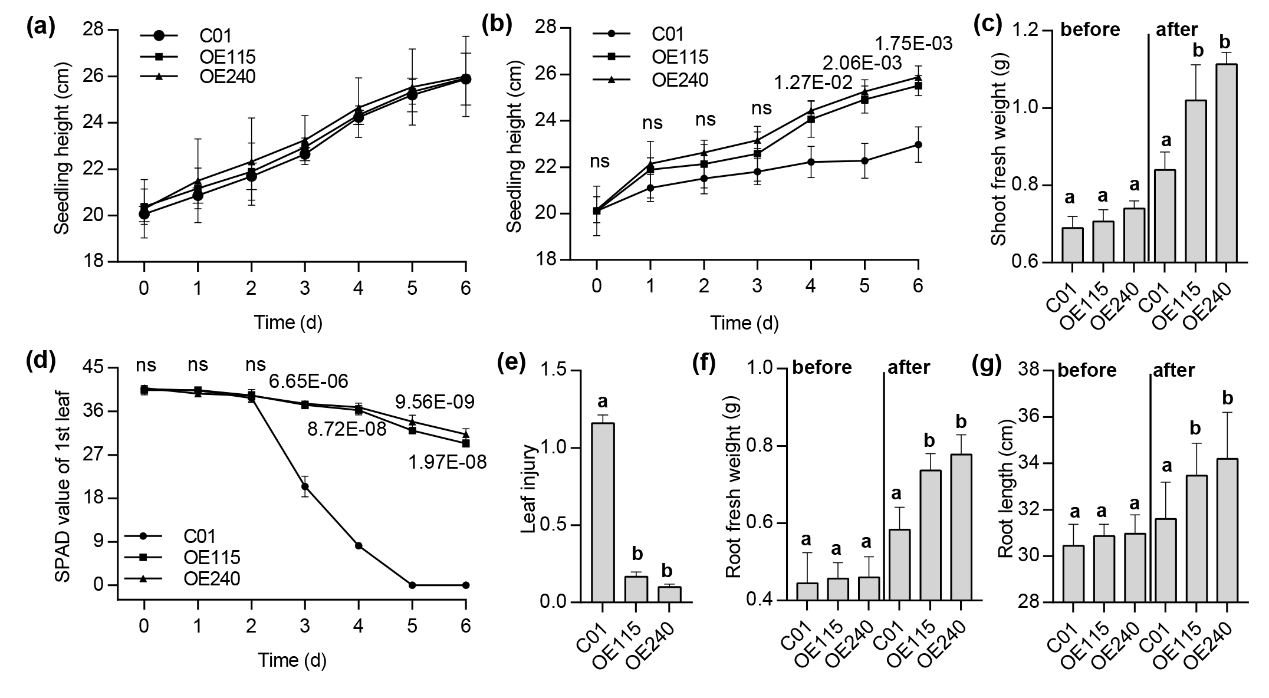


**Fig. S6. Dynamic phenotypes of overexpressing *ZmEREB180* maize lines under waterlogging stress.** The overexpression *ZmEREB180* maize lines (OE115 and OE240) and C01 were subjected to waterlogging stress or growth under normal condition at second leaf seedling stage. (a) The dynamic seedling height of C01, OE115 and OE240 under normal condition. (b) The dynamic seedling height of C01, OE115 and OE240 under waterlogging stress. (d) The dynamic SPAD value of 1st leaf of C01, OE115 and OE240 under waterlogging stress. (c, f-g) The phenotypic difference of the shoot fresh weight, root fresh weight and root length of C01, OE115 and OE240 before and after 6 d waterlogging stress, respectively. (e) The phenotypic difference of leaf injury of C01, OE115 and OE240 after 6 d waterlogging stress. C01 is a transgenic receptor, OE115 and OE240 are two independent transgenic lines. Data represent means ± SD of three independent replicates. The letters above column indicates a statistically significant difference for the data of C01 compared to transgenic lines at different time intervals of waterlogging stress.


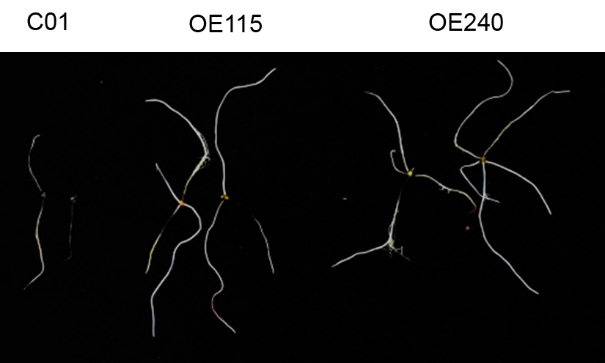


**Fig. S7. Characteristics of adventitious roots of overexpressing *ZmEREB180* maize lines under waterlogging stress.** C01 is a transgenic receptor, OE115 and OE240 are two independent transgenic lines.


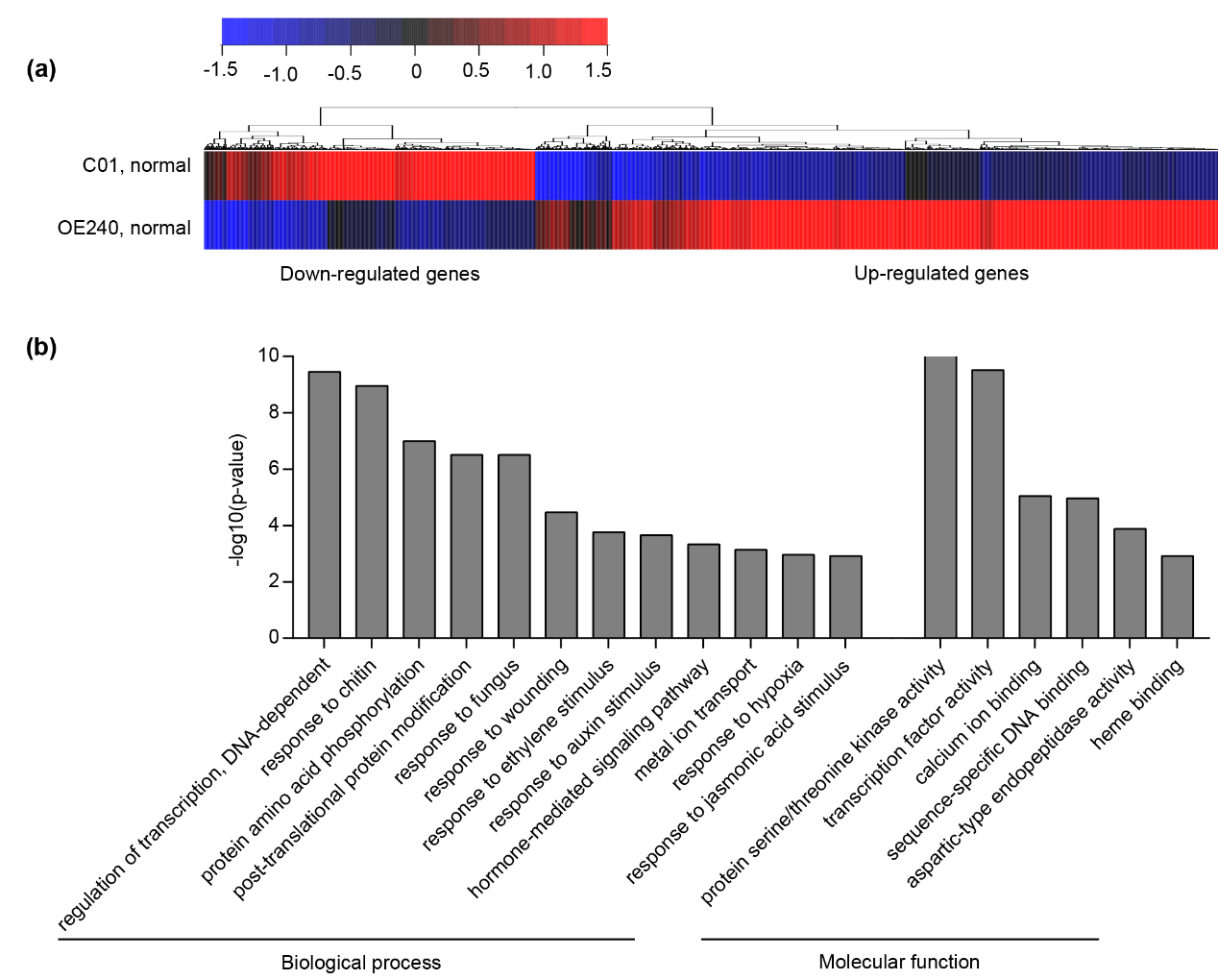


**Fig. S8.** **Transcriptomic analysis of overexpressing *ZmEREB180* maize lines under normal conditions.** (a) Hierarchical clustering of differentially expressed genes in OE240 relative to C01 plants. (b) Significantly enriched GO terms of up-regulated genes in OE240. C01 is a transgenic receptor, OE240 is an independent transgenic line.
